# Building Energy Storage Panel Based on Paraffin/Expanded Perlite: Preparation and Thermal Performance Study

**DOI:** 10.3390/ma9020070

**Published:** 2016-01-25

**Authors:** Xiangfei Kong, Yuliang Zhong, Xian Rong, Chunhua Min, Chengying Qi

**Affiliations:** 1 School of Energy and Environmental Engineering, Hebei University of Technology, Tianjin 300401, China; zylzhongyuliang@gmail.com (Y.Z.); chmin@hebut.edu.cn (C.M.); qicy@hebut.edu.cn (C.Q.); 2 School of Civil Engineering, Hebei University of Technology, Tianjin 300401, China; xrong@hebut.edu.cn

**Keywords:** building energy storage panel, phase change material particle, vacuum absorption, surface film coating, expanded perlite, paraffin

## Abstract

This study is focused on the preparation and performance of a building energy storage panel (BESP). The BESP was fabricated through a mold pressing method based on phase change material particle (PCMP), which was prepared in two steps: vacuum absorption and surface film coating. Firstly, phase change material (PCM) was incorporated into expanded perlite (EP) through a vacuum absorption method to obtain composite PCM; secondly, the composite PCM was immersed into the mixture of colloidal silica and organic acrylate, and then it was taken out and dried naturally. A series of experiments, including differential scanning calorimeter (DSC), scanning electron microscope (SEM), best matching test, and durability test, have been conducted to characterize and analyze the thermophysical property and reliability of PCMP. Additionally, the thermal performance of BESP was studied through a dynamic thermal property test. The results have showed that: (1) the surface film coating procedure can effectively solve the leakage problem of composite phase change material prepared by vacuum impregnation; (2) the optimum adsorption ratio for paraffin and EP was 52.5:47.5 in mass fraction, and the PCMP has good thermal properties, stability, and durability; and (3) in the process of dynamic thermal performance test, BESP have low temperature variation, significant temperature lagging, and large heat storage ability, which indicated the potential of BESP in the application of building energy efficiency.

## 1. Introduction

With the improvement of economic conditions and peoples’ living standards, peoples’ requirements for the thermal environment inside the building have gradually improved [1,2,3], resulting in energy consumption rapidly growing for the air conditioning and heating supply [4,5]. Phase change material (PCM) incorporated with the building envelope for thermal storage has received increasing attention in the building energy efficiency field [6,7]. On the one hand, PCM can increase the thermal inertia of the building envelope and reduce heat loss through latent heat storing to achieve building energy efficiency; on the other hand, PCM can decrease the temperature fluctuation in the building by repeating its thermal absorbing and releasing circulation, for enhancing indoor thermal comfort.

In early research, a sample method was adopted to study the PCM used in buildings, which refers to the liquid PCMs or solid PCM powders that were directly incorporated into the building envelope materials, such as gypsum, mortar and concrete, *etc.* [8,9]. However, PCM was very easy to leak out from building materials, resulting in operational difficulty in practical application [8]. For improving incorporation of PCM into building materials, the method of liquid PCM immersion into shaped building materials (bricks, gypsum boards and concrete slabs, *etc.*) has emerged [10,11,12,13]. Although the immersion method has improved the performance of the direct incorporation method, the leakage of liquid PCM still occurred, especially after repeated cycles [8]. In addition, leakage of PCM can reduce the total amount of PCM used in building, and the mechanical property of construction can also be affected by the interaction between leaked PCM and cement mortars [14]. Therefore, to overcome these issues, composite PCMs with encapsulation techniques, inclusive of microencapsulated PCM and form-stable PCM, have been extensively and deeply studied in recent years.

Microencapsulated PCM can finish the melting and freezing cycle of PCM in the capsule shell with no liquid PCM oozing [8] and, thus, it was generally incorporated into concrete, cement mortar, and gypsum board [15,16,17,18]. However, some incompatibility problems with the building materials have also been experienced. Excessive microencapsulated PCMs can lead to an intensity of building materials decreased and, meanwhile, insufficient microencapsulated PCMs will not achieve the desired effect of thermal storage [19]. Additionally, several capsule shells of microencapsulated PCMs were destroyed in the blending process into concrete or cement mortar, resulting in their stability reducing [20].

The form-stable PCM, which was obtained through PCMs incorporated into supporting materials, has been attracting increasing attention due to its ability to keep the shape of PCM stabilized in the phase change process [8,21]. The greatest advantage of form-stable PCM is that it does not need additional containers or shells to encapsulate PCM and thus form-stable PCM can be incorporated into the building envelope with a simple and convenient implementation. Considering these good points, many form-stable PCMs have been prepared and analyzed in the last two decades.

The components and fabrication method of the form-stable PCM are the important research area in literatures. The organic PCMs such as paraffin, fatty acids and eutectic mixtures of binary PCMs are always used for form-stable PCMs due to their stable thermal performance, high latent heat, and low sub-cooling [22,23,24]. Building materials with a large amount of micropores in their internal structures, such as vermiculite, expanded perlite, diatomite, gypsum, and natural clay, *etc.*, were generally adopted to be the supporting material in form-stable PCM [25,26,27,28]. Thus, the vacuum adsorption was used in incorporation of PCM into supporting material for achieving a better stabilization compared to that of the direct immersion method [14,27,29].

Another research area focused on is the property improvement of form-stable PCM. For improving heat conductivity, addition agents inclusive of expanded graphite, metal powder, and carbon fiber have been mixed in the preparations [30,31] and, thus, the heat transfer performance of form-stable PCM was enhanced. Although the stability and thermal property have been studied and improved in the recent research, an undesired problem of melted PCM leaking from porous materials has been reported. In [32], the fact that paraffin leaked from the PCM cement composites has been presented and, thus, to overcome this shortcoming, the surfaces of diatomite/paraffin composites were modified by using a kind of coating material (Aerosil^®^ R202, Evonik, Herne, Germany) with high surface energy. Li *et al.* have addressed the leakage of form-stable PCM has reduced the thermal performance and workability of cementitious PCM composites, and they proposed that modifiers of hydrophobic fumed silica and precipitated silica using into form-stable PCM can prevent the leakage problem [33]. Ramakrishnan *et al.* have also mentioned the leakage problem for composite PCMs, and in their research a kind of hydrophobic coated expanded perlite (EP) was used to prevent the leakage problem [14]. Sun and Wu have attempted to define the maximum leakage that was acceptable for form-stable PCM using in buildings [34]. Kheradmand *et al.* have indicated that the issue concerning PCMs leakage from porous material should be focused on and, thus, they proposed that PCM composites (impregnated/encased PCMs in lightweight aggregates) were surface coated with waterproofing solutions to solve the liquid PCM leakage problem [35]. From the above research, it can be concluded that the composite PCMs supported by porous materials require a protection to prevent melted PCM leaking.

In this study, a surface protective layer obtained by composite PCM particle immersing into the mixture of colloidal silica and organic acrylate, so as to solve the leakage problem. The composite PCM particle was prepared though vacuum absorption method to achieve the maximum PCM absorption capacity. It should be noted that with no other building materials mixing, such as cements and mortars, the energy storage panel was developed by composite PCM particles and a small amount of adhesives, which can enhance the percentage of PCMs and, thus, achieve a considerable thermal storage capacity. Additionally, related property studies of PCM particle and energy storage panels for the building application were also conducted.

## 2. Experimental

### 2.1. Phase Change Material

**Figure 1:**
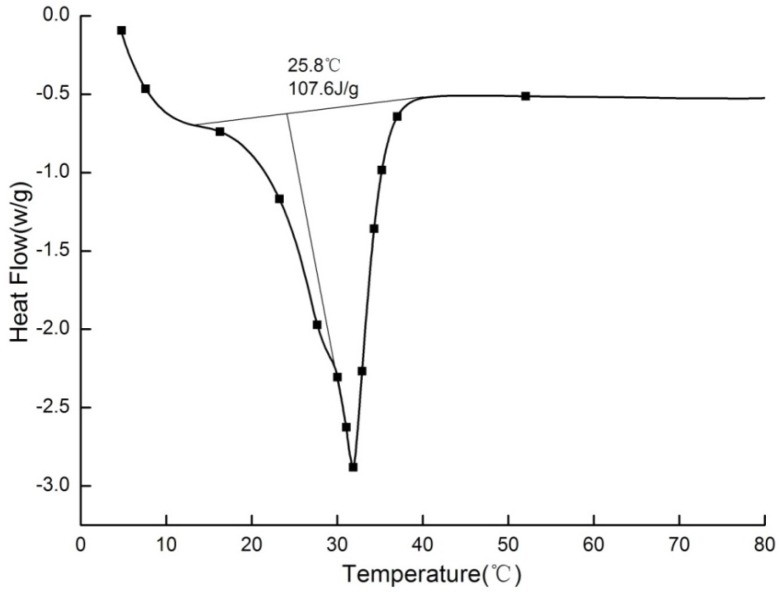
Differential scanning calorimeter (DSC) curves of 25# paraffin.

Paraffin is an organic PCM with the stable performance of non-toxic and non-corrosive and low cost. 25# paraffin purchased from Sinopec Nanyang LTD (Nanyang, China) was chosen to use in this study. As shown in Figure 1, the phase change point is 25.8 °C, which is within the range of indoor thermal comfort [36].The latent heat is 107.6 J/g, which is also suitable for building energy storage [37].

### 2.2. Preparation of PCMP

#### 2.2.1. Support Material

The support material in building energy storage panel (BESP) not only should support the phase change material particles (PCMPs), but also encapsulate PCMs. The expanded perlite, a common building material, has certain mechanical performance and has been widely used in the building of thermal insulation [38,39]. Additionally, it can firmly contain PCM by its porous properties. Therefore, expanded perlite with particle size distribution of 4–6 mm was used to be the support material in this study, and the specific physical parameters are shown in Table 1.

**Table 1:** Property of expanded perlite.

Material	EP
Weight (kg/m^3^)	400
Thermal conductivity (W/(m·K))	0.05
Regenerative coefficient (W/m^2^·K)	2.35
Heat transfer coefficient (W/m^2^·K)	2.45
Specific heat (W·h/(kg·K))	1.17
BET surface area (m^2^/g)	2.68

#### 2.2.2. Preparation Process of PCMP

The preparation of PCMP in this study included two stages: the PCM was absorbed into support material by vacuum absorption method, in order to obtain the composite phase change material; and then, PCMP was prepared by composite phase change material coated with film, so as to guarantee no leakage for PCMP.

**Figure 2:**
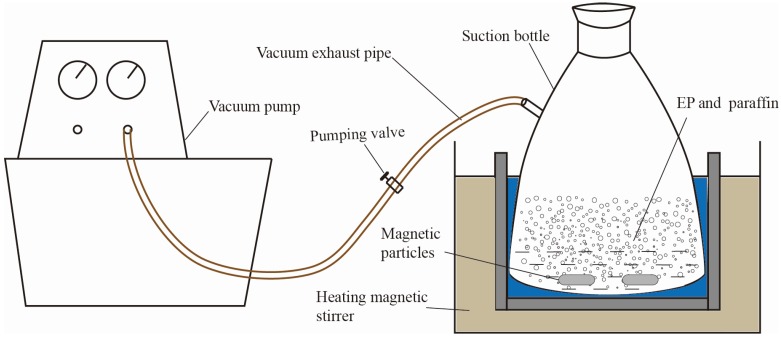
Vacuum absorption experimental sketch.

Figure 2 has shown the process of prepared PCMP by using vacuum absorption, and the detailed procedure was presented as the following.

**Figure 3:**
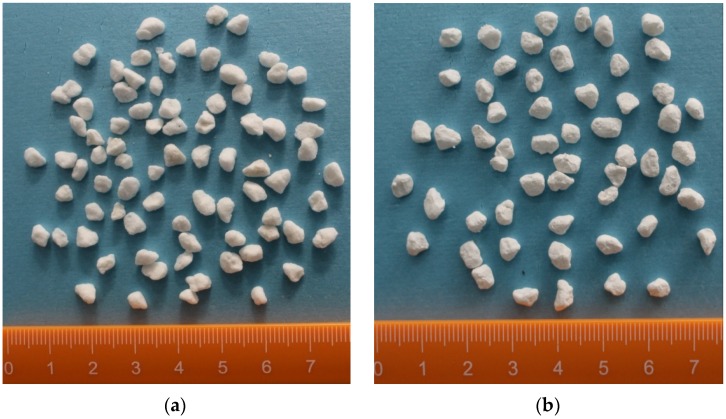
Pictures of composite phase change material (PCM) without surface film coating (**a**) and phase change material particle (PCMP) (**b**).

1. Expanded perlite has been dried in a drying cabinet for 5 h, at first. Then, a certain quality of paraffin, expanded perlite and magnetic particles were put into a suction bottle, and then the vacuum pump was started, in order to keep the internal pressure of the suction bottle to be 0.5 MPa.
2. 30 min later, the heating magnetic stirrer was opened with the heating temperature of 80 °C. Then, the inside pressure of suction flask was further evacuated to 0.01 MPa. 2 h later, the vacuum absorption process was finished.
3. The vacuum pump was closed and, meanwhile, the piston of the flask was removed. The paraffin can be further absorbed into the micropores of expand perlite, especially the residual paraffin in the surface of expanded perlite, under the driving force which was generated from the pressure change (from 0.01 MPa to atmospheric pressure).
4. 1 h later, the heating magnetic stirrer was closed, and then paraffin/expanded perlite in the suction flask were naturally cooled. Finally, the preparation of composite phase change material was accomplished, and the real composite phase change material can be seen in Figure 3a.

Theoretically, PCM can be encapsulated in the support material after vacuum adsorption for the capillary force and surface tension with the micropores in the support material. However, a small amount of liquid PCMs can be leaked out from support material, due to the volume change in phase transition process [14,32,33,34]. Additionally, some kinds of PCMs have a peculiar smell, such as fatty acids. To overcome these shortcomings, a process of film coating for the prepared composite phase change material has been studied.

When the vacuum absorption process is finished, the second stage-coating film process can be started. The composite phase change material was immersed into the mixer of colloidal silica and organic acrylic. After 30 min, the composite phase change material was taken out from the mixture and naturally dried. Finally, a thin film can be created around the surface of composite phase change material to overcome the leakage problem, and the preparation of PCMP was also completed. The real PCMPs were showed in Figure 3b.

### 2.3. Preparation of BESP

**Figure 4:**
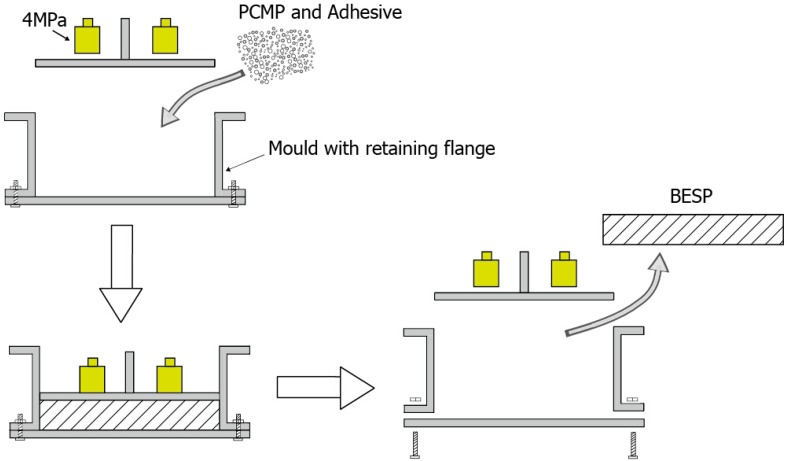
Schematic diagram of the fabricating process of the energy storage panel.

The studied BESP was constituted of PCMP and adhesive (styrene acrylic emulsion in this study) with a certain proportion of the two materials. The mold pressing method was conducted in the BESP preparation, as shown in Figure 4, and the specific steps are as follows:

1. PCMP and styrene acrylic emulsion were mixed and stirred in the mass ratio of 8:1.
2. When they were mixed well, they were put into the mold that has the retaining flanges.
3. The mixture of PCMP and styrene acrylic emulsion were distributed evenly and pressed with the pressure of 4 MPa to maintain the compactness.
4. After a time period of 12 h, the shape of BESP was stabilized.
5. Finally, BESP was successfully prepared, and was fetched out from the disassembled mold.

**Figure 5:**
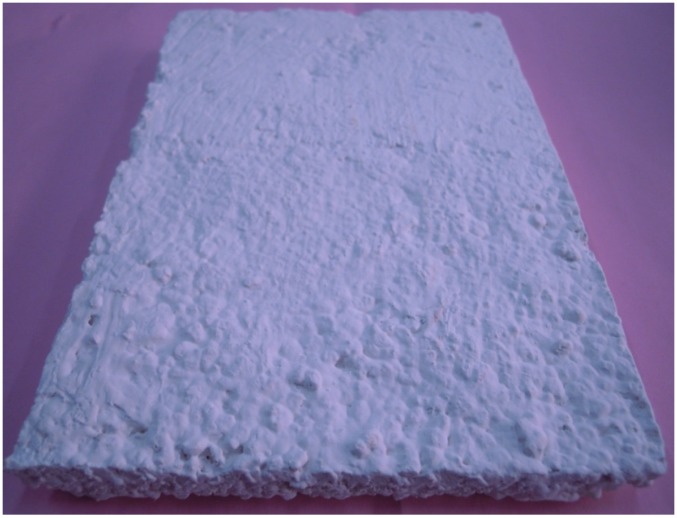
Energy storage panel sample.

In Figure 5, the finished BESP sample has been presented. It can be seen that BESP has a smooth appearance and ready for use in the building envelope.

### 2.4. Leakage Test 

Before the leakage test, a series of PCMPs with different proportions of paraffin and expanded perlite were prepared. Then, a diffusion-oozing circle test proposed by Ma *et al.* [40] was used to conduct the leakage test. The diffusion-oozing circle test is that a standard circle written in a filter paper was used to determine the leak performance of the composite PCMs. In this test, the diameter of standard circle was to be 30 mm, and a certain quality of PCMPs was uniformly distributed into the standard circle. Then, in order to ensure the paraffin completely melted, PCMPs were kept at the temperature of 50 °C for 5 h by a flat heater. When finishing the heating process, PCMPs were removed from the filter paper, and the leakage circle of melted paraffin was measured.

### 2.5. Thermal Property Test 

High thermal property is an important index for PCM application research [27]. In this study, the researched BESP thermal properties included the phase change point, latent heat, and heat conductivity coefficient. The phase change point and latent heat were determined by differential scanning calorimeter (DSC) (DSC2910 model, TA Instruments Company, New Castle, DE, USA). The test sample of DSC was 0.5 mg, which was taken from inside of BESP. Within a nitrogen (N_2_) atmosphere, the sample has been tested by DSC over the range of 0–50 °C at a heating rate of 5 °C/min. In addition, the accuracy of DSC was ±0.1 °C and ±1% for temperature and latent heat, respectively. A heat conductivity coefficient of BESP was measured by a bench-top thermal conductivity instrument (HFM436 model, NETZSCH, Selb, Germany) with the accuracy of ±3%.

### 2.6. Microstructure Observation and Mechanical Property Tests

Scanning electron microscope (SEM) was used to observe the microstructure of BESP and expanded perlite. The SEM pictures were used to analyze the performance of paraffin incorporated into micropores of expanded perlite.

In mechanical property test, the compressive strength of BESP was measured by an electronic universal testing machine (WDW-10, Wintures Testing Technology Co. Ltd., Jinan, China), and the accuracy of this test machine was 20 N. Additionally, the test was started when the styrene acrylic emulsion in BESP was completely solidified.

### 2.7. Durability Test

The melting–freezing cycle was speedily completed by circulating a water bath for the test sample. 1000 cycles were conducted, and the sample after the last cycle was tested by DSC for comparison with it before the cycles. The data of the two DSC tests were compared, in order to determine the durability of BESP in long-time using.

### 2.8. Dynamic Thermal Performance Test

**Figure 6:**
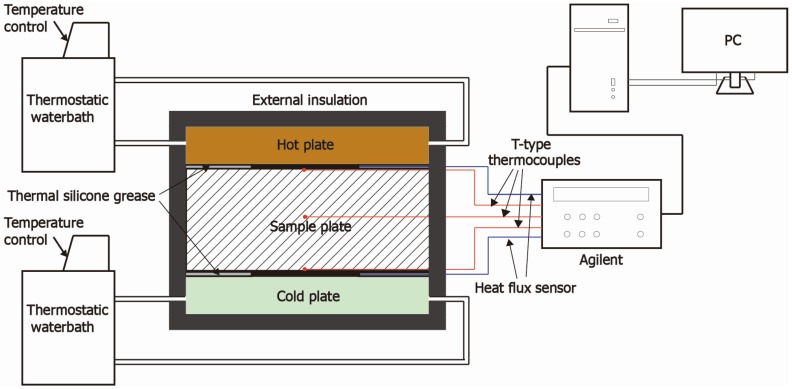
Schematic diagram of dynamic thermal performance test.

A dynamic thermal performance test, as shown in the Figure 6, was conducted for verifying the BESP dynamic thermal behavior, which refers to the temperature response and heat-storage characteristics. The thermal parameters were monitored by T-type thermocouples for temperatures and thermal flux sensors for heat flows, respectively. The data were recorded by an Agilent data logger and transmitted to a personal computer terminal. The detailed information of instruments was listed in Table 2.

**Table 2:** Instrument details.

Instruments	Model	Quantity	Accuracy	Operation Range
Thermocouple	T-type	3	≤±0.4 °C	−35–100 °C
Thermal flux sensor	WYP	2	≤5%	−20–100 °C
Data logger	Agilent 34972A	1	≤0.0041%	-
Thermostatic waterbath	Julabo F-12	2	≤±0.03 °C	−20–100 °C
Hot/cold plate	Manufactured by aluminum sheet; 20 × 20 × 10 mm^3^	2	-	-
PC terminal	Lenovo ThinkStation P300	1	-	-

Two hollow plates connected with two thermostatic water baths were used as dynamic heat and cold sources, respectively, through adjusting the water temperatures of the water baths. A sandwich structure (hot plate-sample-cold plate) was adopted in the testing part, and it was encapsulated with rubber insulation cotton to avoid the external disturbance. Two thermocouples and thermal flux sensors were installed on the hot/cold surfaces of testing sample, respectively, and a thermocouple was embedded into the internal of samples. Additionally, the adjacent surfaces in the sandwich structure were smeared with thermal silicone grease to eliminate the influence of thermal contact resistances and ensure the measurement accuracy of heat across the sample [41].

**Figure 7:**
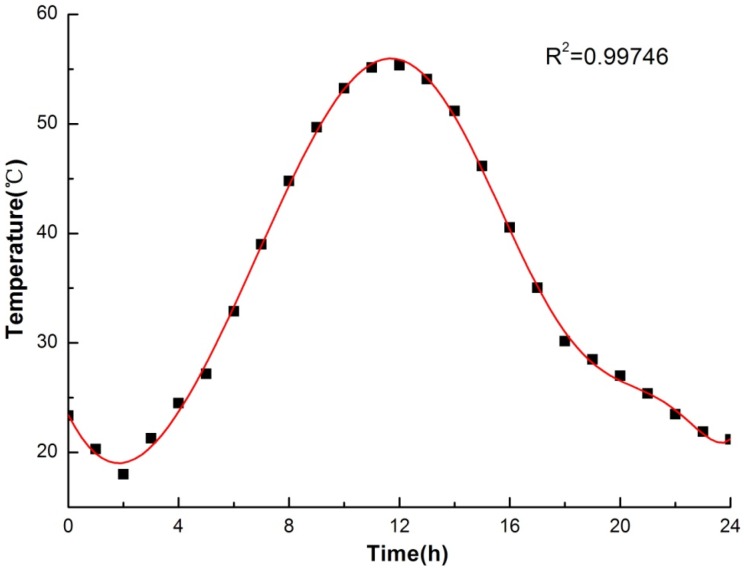
Fitting curve of typical meteorological data and time.

The dynamic operating temperature of the hot plate was inputted according to the Equation (1), which has been obtained through 24-h typical meteorological data [42] of China cold-zone fitting with time and, meanwhile, the temperature of the cold plate was maintained to be 26 °C. The fitting curve was showed in Figure 7.

(1) $$\begin{array}{c} T=11.7643+16.6662\tau -13.1061\tau^{2}+4.9722\tau^{3}-1.0097\tau^{4}+0.1251\tau^{5}\\ -0.0098\tau^{6}+0.0005\tau^{7}-1.4847{\text{e}}^{-0.005}\tau^{8}+2.4704{\text{e}}^{-0.007}\tau^{9}-1.7251{\text{e}}^{-0.009}\tau^{10} \end{array}$$

where T and τ represent temperature (°C) and time (h), respectively; R-squared value is 0.99746. It should be noted that the temperature (*T*) in Equation (1) was the solar-air temperature, which has been ascertained through Equation (2):

(2) $$T=T_{\text{air}}+\frac{aI}{\alpha_{\text{out}}}$$

where *T*_air_ and *I* are the outdoor air temperature (°C) and solar radiation intensity (W/m^2^), respectively; meanwhile, *a* and *α*_out_ represent absorption factor of solar radiation convective and heat transfer coefficient (W/(m^2^·°C)), respectively. Both *T*_air_ and *I* are obtained from the typical meteorological data in [42].

## 3. Results and Discussion

### 3.1. Leakage Analysis

According to [40], η can be used to characteristic the leakage in diffusion-oozing circle test. It is obtained by Equation (3):

(3) $$\eta =\frac{D_{\text{LK}}}{D_{\text{SD}}}\times 100\%$$

where *D* is the diameter of circle, and subscripts of LK and SD represent the leakage circle and standard circle, respectively.

Seven samples with different proportions of paraffin and EP have been tested for leakage, and the results were listed in Table 3. It can be found that the leakage become severe with the mass percentage rising of paraffin and η is also increased. A certain value of η, 115% [34], is considered to be the maximum value, in which the leakage of porous composite PCM is acceptable and safe. Thereby, the paraffin percentage of 52.5 wt % was confirmed to be the most appropriate adsorbing capacity of composite PCM in this study.

**Figure 8:**
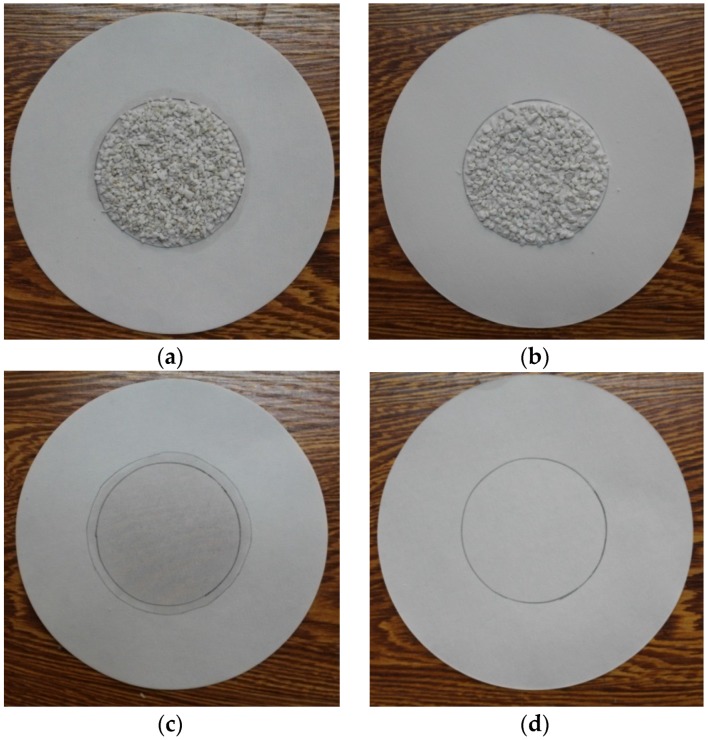
Comparison of leakage performance for with and without coating film: (**a**,**c**) for Sample 5 and (**b**,**d**) for Sample 8.

The last sample (Sample 8) is the composite PCM with coating film. Both PCMs in surface and porous EP were encapsulated into the film in Sample 8, so the leakage of melted PCM was avoided, and the comparison of leakage performance for Sample 5 and Sample 8 was shown in Figure 8. The result that η value of Sample 8 was 100% has indicated the high-quality effect of the coating film in leakage prevention. A similar method to prevent the leakage problem of liquid PCM was also reported in [35], in which several waterproofing materials, such as Sikalastic-490T and Weber Dry Lastic, were chosen to be the surface coating materials for PCM/lightweight aggregates composites, and the good effect on overcoming the PCM leakage problem was experimentally proven. Therefore, the coating film method has the application potential for the preparation of PCM/granular porous material composites.

**Table 3:** Leakage test results.

Samples	Proportion (wt %)	Diameter of Leakage Circle (mm)	Diameter of Standard Circle (mm)	*η*
	Paraffin:EP			
1	35:65	31.50	30.00	105.00
2	40:60	33.01		110.03
3	45:65	33.90		113.00
4	50:50	33.99		113.30
5	52.5:47.5	34.08		113.60
6	55:45	34.77		115.90
7	60:40	35.88		119.60
8	52.5:47.5 (coating film)	30.00		100.00

### 3.2. Thermal Property Analysis

**Figure 9:**
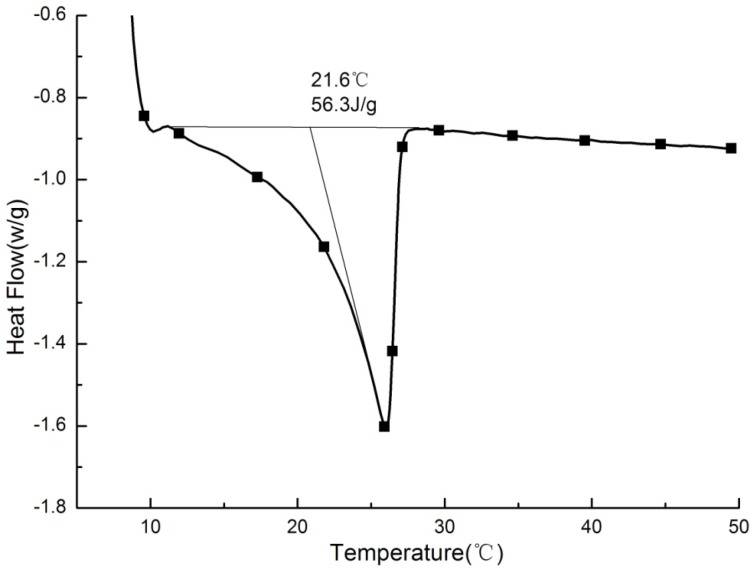
DSC curves of PCMP.

Heat conductivity coefficient of PCMP was determined to be 0.057 W/(m·K) in 30 °C and 0.054 W/(m·K) in 10 °C. Figure 9 has shown the DSC test result of PCMP. Only one distinct peak in the DSC curve indicated a more stable performance in the process of phase change. The phase change point of PCMP was 21.6 °C, with a 4.2 °C difference compared to that of pure paraffin. The fact of the phase change point shift is due to the interaction between paraffin molecules and micropore interfaces of EP. For latent heat, it was found to be 56.3 J/g for PCMP, through calculating area integration at DSC peak curve. The latent-heat ratio of PCMP to pure paraffin was 52.32%, which was closed to paraffin mass percent of 52.5%. Additionally, Table 4 has compared phase change properties of PCMP to that of some composite PCMs with similar phase change point range. It is noteworthy that the prepared PCMP in this study has a high thermal property and significant opportunity in building use.

**Table 4:** Comparison of phase change property of PCMP with that of some composite PCMs in references.

Composite PCM	Phase Change Point (°C)	Latent Heat (J/g)	References
Capric–lauric acid/gypsum	19.1	35.2	[11]
Capric–palmitic acid/gypsum	22.9	42.5	[25]
Capric–stearic acid/gypsum	23.8	49	[26]
Capric–palmitic acid/Vermiculite	23.5	72.1	[27]
Methyl palmitate–stearate/wallboard	22.5	41.1	[43]
25# Paraffin/EP	21.6	56.3	This research

### 3.3. Microstructure and Mechanical Property Analyses

**Figure 10:**
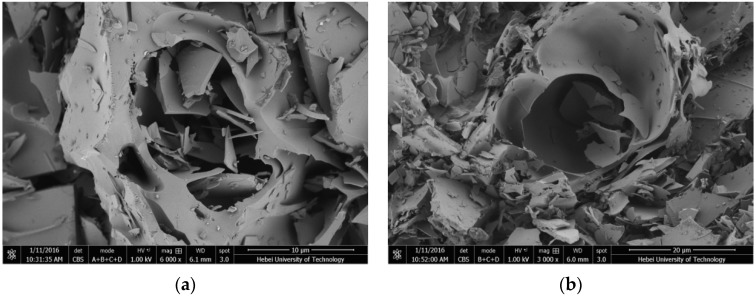
SEM photographs of EP with different magnification: (**a**) 10 μm and (**b**) 20 μm.

In SEM picture of Figure 10, EP has obvious crack surface layer and three dimensional framework structures within lots of micropores. These pores used to absorb paraffin are numerous with thin wall, unfixed shapes, and size range of a few micrometers to several hundred micrometers. What is more, due to the function of capillary action and surface tension, the porous structure can contain and constraint the molecules of liquid paraffin. Thereby, this special structure provides adsorption effect for PCM.

**Figure 11:**
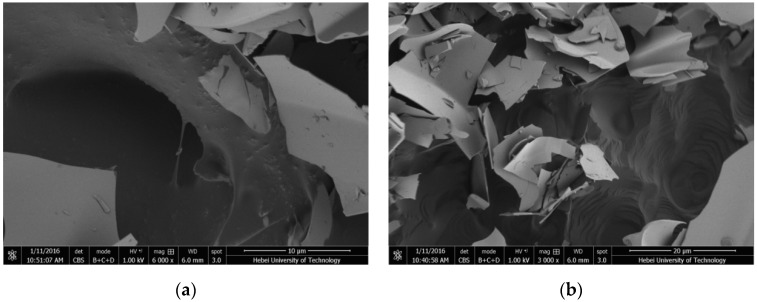
SEM photographs of PCMP after surface film coating with different magnification: (**a**) 10 μm and (**b**) 20 μm.

The SEM picture of PCMP is shown in Figure 11. It is obvious that the appearance of PCMP was different compared with that of EP. The paraffin with smooth surface and black color was contained by the porous and laminated structure of EP (white color). It has been illustrated that EP with a three-dimensional net micropore structure can firmly absorb paraffin. Additionally, coating film in the outer surface can further encapsulate composite PCM particle. Thus, the microstructure of PCMP was stable and tightness.

In the mechanical property test of BESP samples, their average compressive strength was 4.61 N/mm^2^, which was about 2 N/mm^2^ higher than some EP insulation boards composed of plaster, EP, and water [44]. Furthermore, the compressive strength of BESP was lower than that of some EP concrete boards. After concrete maintenance, the compressive strength can reach to 7.53 N/mm^2^ for a kind of EP/PCM concrete boards, but the cement mortar accounted for 70% of total mass and, thus, the latent heat was decreased [14]. A deep study on the type and mass ratio for adhesives and their influence on the adhesive strength and thermal performance for BESP will be launched in the next work, so as to enhance the mechanical and thermal property of BESP.

### 3.4. Durability Analysis

**Figure 12:**
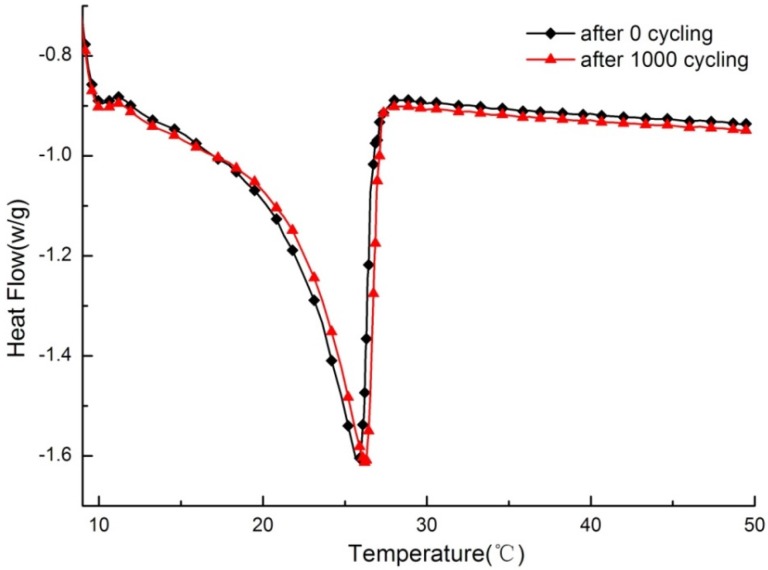
DSC curve of PCMP before and after thermal cycling.

Figure 12 shows the DSC curve comparing test samples before and after a melting–freezing cycle of 1000 times, respectively. For the whole variation tendency of thermal properties with increasing temperature, the DSC curve before cycling is consistent with that after thermal cycling. Then, as listed in Table 5, after 1000 cycles, the onset point, peak point, and end point of the DSC curve for PCMP have changed 0.5, 0.2, and −0.3 °C, respectively, followed by corresponding change rates of the three points of 2.31%, 0.77%, and −1.08%, respectively. These small changes can be considered to not be significant in magnitude for PCM applications [25]. Finally, the latent heat has decreased to be 55.8 J/g, which is 0.5 J/g smaller than that before the thermal cycles. The slight decrease was within a reasonable level for a composite PCM [25].

**Figure 13:**
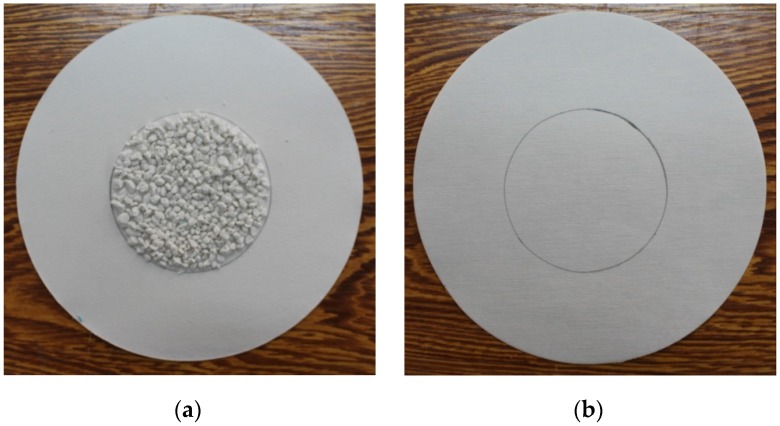
Leakage test for PCMP after 1000 thermal cycles. (**a**) PCMP was in filter paper after thermal cycles; (**b**) PCMP was removed from filter paper after thermal cycles.

In conclusion, the reproducible thermal performance for the test sample has indicated that PCMP has good thermal stability and reliability. Additionally, after leakage tests for the sample with thermal cycles, it was confirmed that PCMP still has no leakage during long-term use, as shown in Figure 13.

**Table 5:** Phase change parameters before and after thermal cycles of PCMP.

Cycle Number	Onset Point of Phase Change (°C)	Peak Point of Phase Change (°C)	End Point of Phase Change (°C)	Latent Heat (J/g)
0	21.6	26.0	27.8	56.3
1000	22.1	26.2	27.5	55.8

### 3.5. Dynamic Thermal Performance Analysis

**Figure 14:**
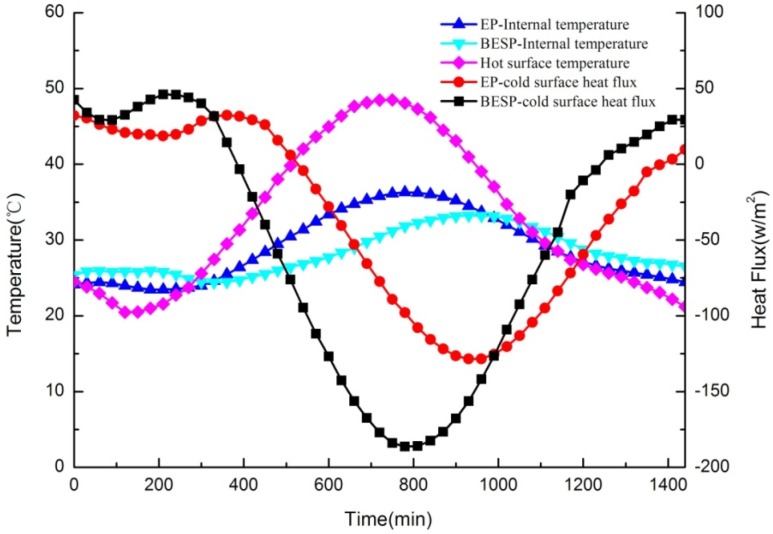
Comparison of dynamic thermal performances for building energy storage panel (BESP) and expanded perlite (EP) panel over 24 h.

In this test, the internal temperatures of test panels were measured to analyze the thermal response and, meanwhile, the heat flux between samples and the cold surface were also monitored to study the thermal storage performance of panels. Furthermore, the hot surface temperature that varied according to the 24-h typical meteorological data in summer represented the outside temperature. The dynamic thermal performances for BESP and EP panels (no paraffin in the EP panel) were compared, as shown in Figure 14, in which three important points can be highlighted as follows:

It was firstly obvious that with the external temperature changing, the temperature fluctuation of BESP was smaller than that of the EP panel, which can be illustrated by the fact that BESP was 3.08 °C lower than the EP panel for the maximum temperatures. This was because the characteristic of PCM with a small temperature range in the melting/freezing process has reduced the large temperature fluctuation. The smoother temperature variation for BESP has indicated that it can enhance the thermal comfort inside the building.

The second observation is that a significant lagging effect of temperature response occurred in BESP. The peak temperature of internal BESP was 180 min later than that of the outside temperature, but the internal peak temperature lagging for EP panel was only 30 min. The larger temperature lagging has shown that BESP had a more prominent thermal inertia, and can decrease the energy consumption of buildings.

Furthermore, the average heat flux across BESP was 17.11 W/m^2^ smaller than that across EP panel, followed by maximum heat flux decrease of 57.76 W/m^2^. The lower heat flux of BESP implied less heat was transmitted through the panel, compared with that of EP panel. This was due to, in the heat transmission process, BESP can store more heat through PCM melting with heat absorption.

## 4. Conclusions

In this study, a novel composite PCM particle (PCMP) with a coating film has been prepared, through paraffin incorporated with EP by vacuum adsorption. With the coating film process completed for PCM particles, the leakage problem in composite PCM has been effectively resolved. Then, a thermal storage panel–BESP was fabricated with PCMPs by a mold pressing method with a certain amount of adhesives. Furthermore, the related properties of PCMP and dynamic thermal performance of BESP have been studied and analyzed, and several conclusions were obtained, as following:

- The best proportions of paraffin and EP in PCMP were ascertained to be 52.5 wt % and 47.5 wt %, respectively, and no liquid paraffin oozed from PCMP in leakage tests, after applying a coating film on PCMP.
- The phase change temperature and latent heat of PCMP were measured to be 21.6 °C and 56.3 J/g, respectively, through DSC.
- There was no significant degradation of thermal properties for PCMP sample after repeating melting and freezing cycles 1000 times.
- A high-quality thermal performance, including small temperature fluctuation, large temperature lagging, and high thermal storage capacity for BESP, was confirmed in a dynamic temperature input test.

BESP, a method of application for PCMP, was developed and its good thermal performance was obtained, however, related other characteristics, such as water-resistance ability and adhesive affection, *etc.*, have not been involved in this study. There is, therefore, the need to complete the BESP property research in future work, in order to achieve the BESP large-scale application in buildings.
